# Improving Brain Creatine Uptake by Klotho Protein Stimulation: Can Diet Hit the Big Time?

**DOI:** 10.3389/fnut.2021.795599

**Published:** 2021-12-23

**Authors:** Sergej M. Ostojic, Dagrun Engeset

**Affiliations:** Department of Nutrition and Public Health, University of Agder, Kristiansand, Norway

**Keywords:** creatine, CT1, brain, Klotho (KL), vitamin D2, curcumin, phosphate-restricted diet, low-calorie high-protein diet

## Introduction

Creatine plays a pivotal role in cellular bioenergetics, acting as a temporal and spatial energy buffer in cells with high and fluctuating energy requirements (1). Jeopardizing delicate creatine homeostasis can be detrimental to many energy-demanding tissues, including the brain. For instance, cerebral creatine hypometabolism accompanies various neurological conditions, including a number of developmental disorders (2, 3), neurodegenerative and cerebrovascular diseases (4, 5), and brain cancer (6). A reduced creatine availability in the brain has been thus recognized as an apposite therapeutic target, and supplying exogenous creatine to compensate for a disease-driven shortfall emerged as a first possible approach. However, early success in animal models of neurological diseases was not corroborated in human trials, with the use of creatine supplementation proved largely disappointing in clinical studies with a number of symptomatic neurological disorders [for a detailed review, see (7)]. A meager delivery of creatine to the brain could be partly due to a low activity/density of creatine transporter (CT1 or SLC6A8), a transmembrane sodium- and chloride-dependent protein that mediates creatine uptake into the target cells (8). For that reason, the upregulation of CT1 function has been identified as an innovative course of action to facilitate creatine uptake, with several exotic agents and routes were cataloged so far, including glucocorticoid-regulated kinases, mammalian target of rapamycin, ammonia, and Klotho protein (9).

## CT1 Stimulation and Klotho Protein

**Figure 1:**
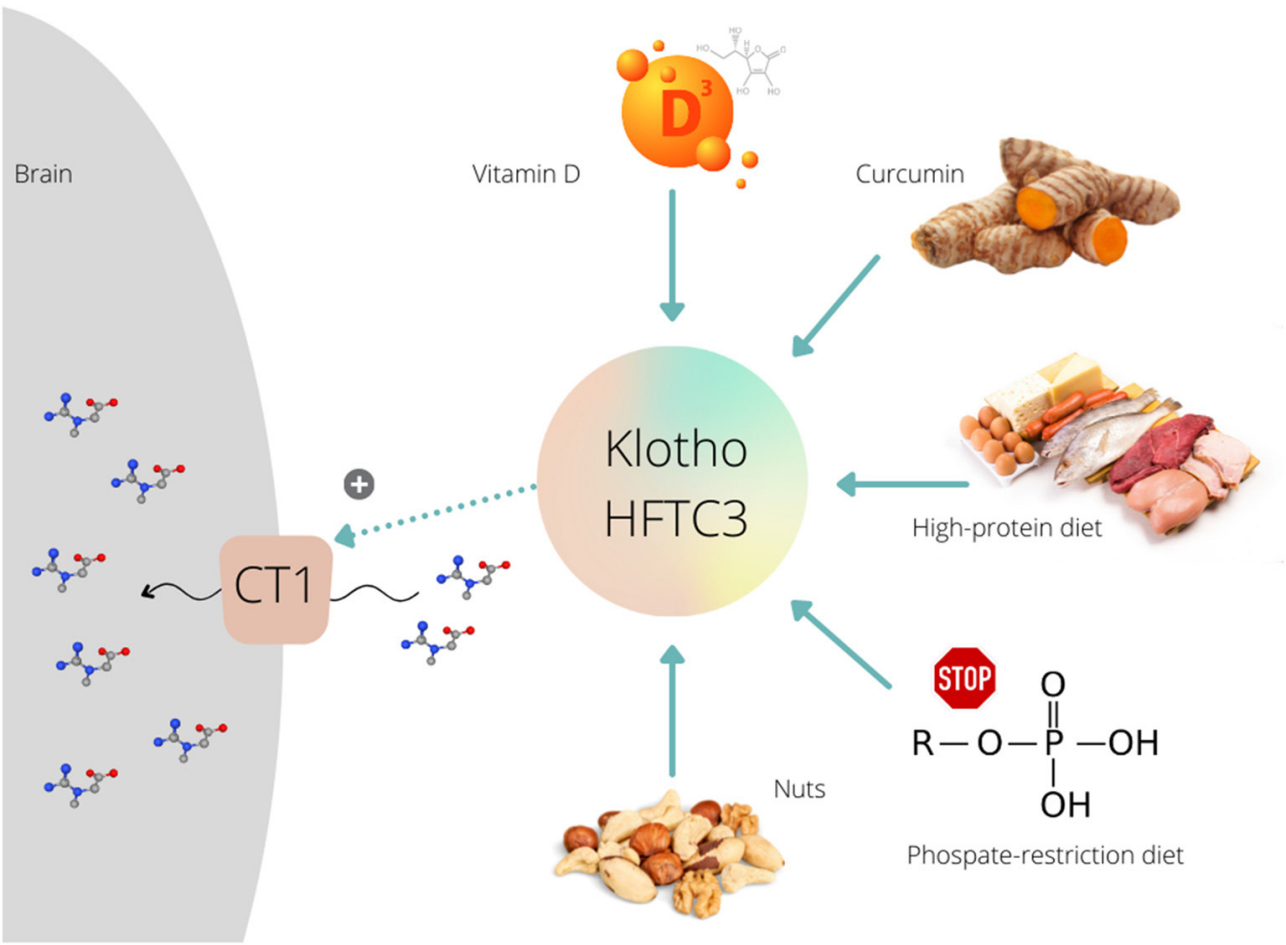
Various dietary interventions upregulate Klotho protein expression/activity that further can stimulate (+) creatine uptake via creatine transporter (CT1) in the brain.

Besides other vehicles, Klotho protein (Clotho; HFTC3) is put forward as a possible stimulator of CT1 function that can uplift creatine allocation to the target tissues. This membrane-bound pleiotropic enzyme (also exists in a circulating form) participates in many metabolic pathways, including calcium-phosphate metabolism, nutrient sensing, and remyelination (10). Klotho is highly expressed in neuronal cells of the cerebral cortex, cerebellum, and spinal cord (11). The role of Klotho in high-phosphate energy metabolism modulation was revealed a few years ago when Amilaji et al. (12) found that the co-expression of Klotho protein increases a creatine-induced current in CT1-expressing cells. The authors reported that the current through CT1 was a function of the extracellular creatine levels, with the maximal creatine-induced current was higher in cells expressing CT1 together with Klotho than in cells expressing CT1 alone (29.5 vs. 20.2 nA). This implies a Klotho-driven upregulation of creatine carriers, presumably by stabilizing the carrier protein in the cell membrane, which likely nominates Klotho protein as a therapeutic proxy to accelerate creatine uptake through CT1. Several methods have been developed lately to raise Klotho levels (13), including a transgenic insertion of the klotho gene, a recombinant Klotho protein administration, or using angiotensin-converting enzyme inhibitor and thiazolidinediones. However, nutrition-related interventions might also be capable of enhancing endogenous Klotho production, and perhaps to unlocks CT1 for brain creatine uptake (Figure 1).

## Klotho Modulation by Diet

Several preclinical studies demonstrated that exposure to vitamin D could modulate the activity of Klotho protein. A Japanese group (14) treated mice with a vitamin D-enriched diet and found increased expression of Klotho; the expression was upregulated and reached maximal levels 8 h after the administration of 1,25-dihydroxyvitamin D3. Vitamin D treatment induced a Klotho mRNA hyper-expression in different mouse renal cell lines (15), and in proximal kidney human cells (16). Curcumin, a polyphenol pigment and principal curcuminoid of turmeric (Curcuma longa), is another dietary agent that can upregulate Klotho protein. Hu et al. (17) demonstrated that curcumin induced Klotho expression in HK-2 human proximal tubule epithelial cell line. Recent studies found that the whole-diet interventions are also effective to intensify Klotho function, with a low-calorie high-protein diet increased Klotho levels in rats' brain (18), while a phosphate-restricted diet improved kidney klotho expression in a mouse model of polycystic kidney disease (19). Interestingly, a positive association was found between nuts portion intake and the Klotho plasma levels in middle-aged sedentary adults (20). These preliminary findings call for a set of proof-of-concept studies where supplemental creatine is co-administered with Klotho-stimulating agents while participants are monitored for changes in brain creatine levels. A recent trial confirmed favorable outcomes of a creatine-vitamin D multi-component supplement for functional performance and upregulation of energy metabolism-related kinases in elderly (21). Yet, no biomarkers of Klotho activation and/or creatine uptake by the brain have been evaluated.

## Conclusion

Although promising, backing up the Klotho activity to improve creatine delivery to the central nervous system requires robust evidence. Basically, whether the Klotho upregulation induced by a specific dietary intervention is followed by a CT1 triggering and augmented creatine uptake in the brain currently remains unknown. Experimental transport/expression models in neurons would help with addressing CT1-Klotho activation in interventional studies, while clinical trials might include monitoring circulating Klotho as a proxy to Klotho stimulation, along with MR spectroscopy to track down changes in brain creatine levels. Future studies require a careful scrutinization of nutritional strategies utilized in terms of the length of intervention and level of exposure, and adjusted for creatine consumed from food. Additional covariates that can influence the outcome of a given trial also include concomitant pathology (such as X-linked creatine transporter deficiency) and nutritional status of participants (e.g., vitamin D deficiency, creatine-free diet), along with other factors (including age, physical exercise, and level of education) that could play important role on CT1 and Klotho protein function and/or creatine homeostasis in the brain. For instance, animal studies suggest sex differences in Klotho protein expression, with the levels were higher in the male mice than in the female mice (22). Klotho function could also be affected by HMG-CoA reductase inhibitors (23), uremia-related compounds (24), or demethylation agents (25). Therefore, accounting for various endogenous and exogenous modulators of Klotho protein should be required for upcoming nutritional trials addressing brain creatine uptake through this pathway. The new knowledge about a possible diet-induced stimulation of Klotho protein opens an exciting opportunity for exploring various nutritional strategies aimed to potentiate cerebral uptake of creatine. This could perhaps bring forth a solution to the many who suffer from acquired or inherited creatine deficits.

## Author Contributions

SO designed and wrote the manuscript and has primary responsibility for the final content. All authors read, revised, and approved the final version of the manuscript.

## Conflict of Interest

SO serves as a member of the Scientific Advisory Board on creatine in health and medicine (AlzChem LLC.). SO owns patent “Sports Supplements Based on Liquid Creatine” at European Patent Office (WO2019150323 A1), and active patent application “Synergistic Creatine” at UK Intellectual Property Office (GB2012773.4). SO has served as a speaker at Abbott Nutrition, a consultant of Allied Beverages Adriatic and IMLEK, and has received research funding related to creatine and/or guanidinoacetic acid from the Serbian Ministry of Education, Science, and Technological Development, Provincial Secretariat for Higher Education and Scientific Research, AlzChem GmbH, KW Pfannenschmidt GmbH, Monster Beverage Corporation, and ThermoLife International LLC. SO does not own stocks and shares in any organization. The remaining author declares that the research was conducted in the absence of any commercial or financial relationships that could be construed as a potential conflict of interest.

## Publisher's Note

All claims expressed in this article are solely those of the authors and do not necessarily represent those of their affiliated organizations, or those of the publisher, the editors and the reviewers. Any product that may be evaluated in this article, or claim that may be made by its manufacturer, is not guaranteed or endorsed by the publisher.
